# ‘Going through the motions’; a rich account of the complexity of the anterior cruciate ligament reconstruction pathway, a UK qualitative study

**DOI:** 10.1136/bmjopen-2023-079468

**Published:** 2024-09-17

**Authors:** Hayley M Carter, David J Beard, Paul Leighton, Fiona Moffatt, Benjamin E Smith, Kate E Webster, Phillipa Logan

**Affiliations:** 1 Physiotherapy Outpatients, University Hospitals of Derby and Burton NHS Foundation Trust, Derby, UK; 2 School of Medicine, University of Nottingham, Nottingham, UK; 3 Surgical Intervention Trials Unit, Botnar Research Centre, NDORMS, University of Oxford, Oxford, UK; 4 School of Health Sciences, University of Nottingham, Nottingham, Nottinghamshire, UK; 5 La Trobe University, Melbourne, Victoria, Australia

**Keywords:** Orthopaedic sports trauma, Adult orthopaedics, Knee

## Abstract

**Abstract:**

**Objectives:**

This study aimed to understand the lived experiences of patients on the anterior cruciate ligament reconstruction (ACLR) pathway up to 3 months before, 3 months after and 1 year after surgery. Study objectives were to explore (1) patient experiences of preoperative and postoperative treatment, (2) views of/involvement in prehabilitation and (3) sources and consistency of healthcare advice.

**Design:**

Semi-structured interviews analysed using reflexive thematic analysis.

**Setting:**

Midlands, England.

**Participants:**

Purposive sample of 18 participants aged 18–45. Three identified as female and 15 as male. Participants’ ethnic origin was white (n=14), Indian (n=2), British Asian (n=1) and Pakistani (n=1). 10 participants were awaiting ACLR, six were 3months postsurgery and two were 1 year postsurgery.

**Results:**

Participants gave a rich account of ACLR pathway experiences discussing negative impacts of the injury, difficulties with navigating the pathway and making decisions about surgery. Interacting with healthcare professionals and managing the variety of resources, advice and opinions were also highlighted as challenges. Participants reflected on their preoperative journey accounting a wide spectrum of expectations and realities of returning to work and physical activity postoperatively. Prehabilitation was perceived to offer an advantage to recovery, mental well-being, injury knowledge, postoperative rehabilitation and supports a faster return to physical activity. Five themes were identified:

1. Injury experience, impact and support.
2. Navigating the treatment pathway.
3. Sense making in the preoperative period.
4. Uncertainty, expectations and reality of the postsurgical period.
5. Balancing resources, advice and opinions.

**Conclusion:**

This study has illuminated patient experiences of the National Health Service (NHS) ACLR pathway, novel to the evidence base.

The results highlight the perceived shortcomings in patient support. They also demonstrate the difficulty patients face when navigating the NHS system, communicating with clinicians, making decisions about treatment and managing conflicting sources of healthcare advice. These problems are more prominent than previously recognised in the literature.

**Registration:**

ClinicalTrials.gov Identifier: NCT05529511.

**STRENGTHS AND LIMITATIONS OF THIS STUDY**

- In terms of strengths, this qualitative interview study gives voice to the experiences of adults who have had or are awaiting anterior cruciate ligament reconstruction in the National Health Service (NHS) in England. The reflexive thematic analysis approach facilitated rich engagement with the data to produce detailed accounts of participant experiences.
- A further strength of the study was the collaboration with the trial steering committee, including patients and stakeholders, during data analysis.
- With regard to limitations, the interview medium varied across participants (face-to-face or virtual) which may have resulted in differing relationships between the participant and researcher which may have impacted on interview data.
- There were a greater number of participants at the preoperative time point which is likely to have resulted in a greater richness of data regarding the preoperative pathway than that of the postoperative pathway (particularly at the 1-year time point where only two participants were interviewed).
- The study population were treated within hospitals in one region of the UK (Midlands), and while it is not the aim of qualitative research to be generalisable, these findings may not represent the experiences of those treated in other UK regions and outside of an NHS setting.

## Introduction

 The median annual incidence of anterior cruciate ligament (ACL) rupture in the general population is 0.03%, equating to approximately 20 200 ruptures each year in the UK.^1^ Once diagnosed, treatment may follow a non-surgical or surgical approach. Surgery rates for ACL injuries increased 12-fold in the UK between 1997 and 2017, with a rate of 24.2 ACL reconstructions (ACLR) per 100 000 of the population.^2^ Rehabilitation prior to surgery is recommended;^3^ however, guidance supporting clinicians in delivering evidence-based practice is limited and thus clinical practice varies widely.^4^ Rehabilitation is also completed postsurgery, although the breadth of research in this area is vast and protocols are consistently reported to be heterogeneous with no consensus on the most clinically effective approach.^5 6^

Patient-centred care is a core ethos of the National Health Service (NHS), outlined in the long-term plan as a key deliverable.^7^ Limited research exists to describe the patient experience of sustaining an ACL injury and navigating the ACLR pathway, and so our ability to deliver patient-centred care is suboptimal. To date, only quantitative measures collected from cohort studies have reported patients’ preoperative expectations of return to sport (RTS) following ACLR.^8 9^ Postoperative perspectives from 6 months to 10 years postsurgery have, however, been explored through semistructured interviews.^10^,^12^ Collectively, these studies reveal that patients have unrealistic preoperative expectations of returning to physical activity postsurgery and are faced with a postoperative rehabilitation burden that requires an unexpected level of commitment, with participants describing a lack of mental preparation for the rehabilitation process that was longer and more intense than expected.^9 11 12^ There is an absence of knowledge to understand participants’ lived experience of this phenomena, particularly prior to surgical intervention. Further, there is a paucity of evidence to understand where patients seek healthcare advice following an ACL rupture diagnosis.

The aim of the study was to understand the patients’ lived experiences of the treatment pathway following a diagnosis of an ACL rupture and agreed surgical management. Study objectives were (1) to explore lived experiences at preoperative and postoperative time points, (2) to explore patients’ views and involvement in prehabilitation and (3) to understand patients’ sources and consistency of healthcare advice prior to surgery.

## Method

Reflexive thematic analysis was chosen to analyse the data, aligning to the lead researchers’ philosophical underpinnings of pragmatism. The text offered by Braun and Clarke^13^ was used to support analysis and the study reported in line with the COnsolidated criteria for REporting Qualitative research checklist (online supplemental file 1).^14^

## Recruitment

We sought to recruit approximately 12 patients at three separate time points (3 months prior to surgery, 3 months after surgery and 1 year after surgery), estimating that this would be sufficient to reach data saturation and mirrored similar research of musculoskeletal conditions.^15^,^18^ We aimed to include a range of participant characteristics including age, sex, physical activity type and level and prehabilitation engagement (detailed in tables1  2).

**Table 1:** Participant characteristics

Participant number	Sex	Time point on ACLR pathway	Prehabilitation(Y/N)	Average number of days physically active preinjury	Average number of days physically active at point of interview	Returned to preinjury activity level (Y/N)
001	M	3 months postoperative	N	4	4	N
002	M	Preoperative	Y	4	3	N
003	M	Preoperative	Y	3	1	N
004	F	Preoperative	Y	3	4	Y
005	M	Preoperative	Y	4	1	N
006	M	Preoperative	Y	4	2	N
007	M	Preoperative	Y	7	7	N
008	M	3 months postoperative	Y	6	7	N
009	M	Preoperative	Y	4	4	N
010	F	Preoperative	Y	3	1	N
011	M	3 months postoperative	N	5	5	N
012	M	3 months postoperative	N	4	4	N
013	M	Preoperative	Y	5	0	Y
014	M	Preoperative	Y	3	2	N
015	F	1 year	N	5	5	N
016	M	3 months postoperative	N	7	6	N
017	M	1 year	N	4	2	N
018	M	3 months postoperative	Y	6	3	N

**Table 2:** Activity types

Activity type	Number of participants engaging in the activity preinjury	Number of participants engaging in the activity at the time of interview
Badminton	1	
Basketball	1	
Cricket	3	
Cycling	1	1
Football	8	
Golf	1	1
Gym (cardiovascular and resistance training)	5	5
Hiking	1	
Indoor cycling	1	1
Judo	1	
Mountain biking	2	
Muscle strength training	7	7
Netball	2	
Road cycling		1
Rugby	3	
Running	3	3
Snowboarding	1	
Squash	1	
Swimming	4	1
Tennis	2	
Volleyball	1	
Wakeboarding	1	
Walking	2	5
Yoga		1

Participants were identified by the clinician in charge of their care and recruited from physiotherapy and orthopaedic waiting lists at the University Hospitals of Derby and Burton NHS Foundation Trust (UHDB). Orthopaedic waiting lists were screened for two lower limb consultants at UHDB and all outpatient musculoskeletal (MSK) physiotherapists working at the Florence Nightingale Community Hospital. All patients had received an MRI and had a consultation with an orthopaedic clinician to determine the extent of concomitant injuries. Clinicians were aware of the study eligibility criteria and highlighted all those appropriate for inclusion in the study. They were subsequently contacted by mail or telephone or introduced in person to the researcher (HC).

Eligibility criteria, shown in table 3, were prescreened by the identifying clinician and then checked prior to consent being gained for participation in the study (HC).

**Table 3:** Eligibility criteria

Inclusion	≥18 years old At one of the three identified time points (3 months prior to surgery, 3 months after surgery and 1 year after surgery)
Exclusion	Concomitant injury requiring surgical intervention that is anticipated to significantly alter the postoperative rehabilitation protocol (eg, meniscal repair requiring a non-weight bearing period) Previous knee surgery to the affected limb Coexisting injuries requiring surgical intervention impacting on ability to participate in preoperative or postoperative rehabilitation Pregnant (as this would affect rehabilitation participation and surgical timings)

## Data collection

Participants were offered the choice of interview location and medium (face-to-face/telephone/video). Eight participants opted for a face-to-face interview conducted in a hospital setting, and the remainder opted for a telephone interview. Interviews were carried out between August and November 2022. Prior to each interview, the researcher (HC) introduced herself as a physiotherapist working at UHDB and as a researcher conducting a PhD at the University of Nottingham. Written consent was taken prior to the interview and recording.

Semistructured interviews were conducted using a topic guide (online supplemental file 2) developed using the literature, research team and patient and public involvement (PPI) input. The researcher maintained a reflexive journal to document the thoughts after each interview, in addition to revisiting initial interview recordings to review interview and questioning technique. This practice helped to identify areas where the researcher was hesitant to prompt for further clarification and supported deeper exploration of concepts in later interviews. It also acted as a basis for discussion during research group meetings and allowed for participant characteristics to be recognised ensuring recruitment was responsive to data collection.

## Data analysis

Audio files were transcribed by a third-party vendor. Transcriptions were reviewed against the audio recording by HC for accuracy and were read several times to support data immersion. Transcripts were coded by HC with code generation discussed among the research team. 75% of codes were generated by interview 6, and 100% were reached by interview 16, offering reassurance that no new codes were arising in the latter stages of recruitment.

**Figure 1:**
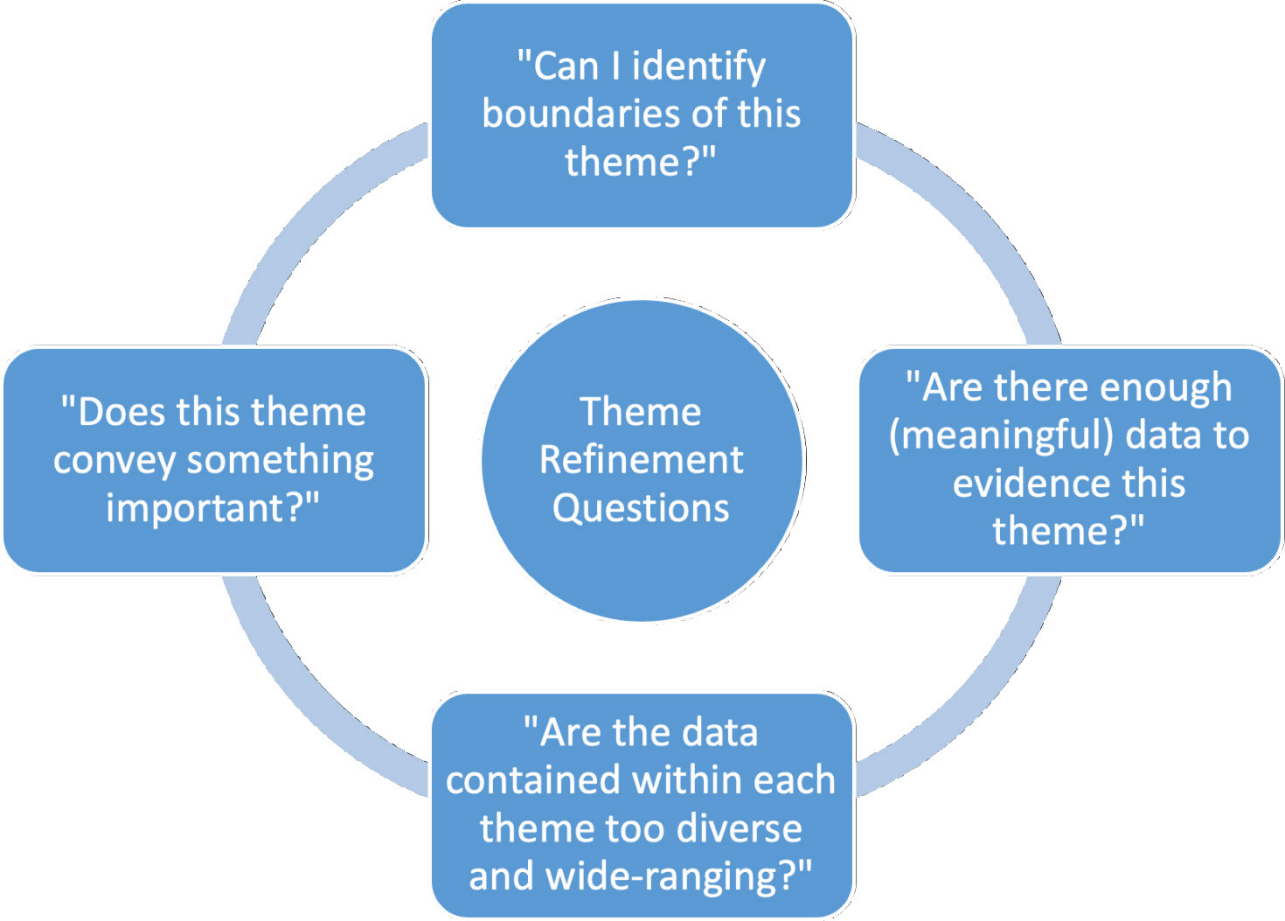
Braun and Clarke questions used to support theme refinement.

The codes were organised into five themes, aligning with the research objectives. These themes and supporting extracts were discussed among the research team and were felt to offer a rich and clear insight of the data, individually representing an organising concept while contributing to the narrative of other themes and thus the entire dataset. The questions, shown in figure 1, from Braun and Clarke were used to support the refinement of each theme.

Data were organised and coded in NVivo V.12. Themes were developed in Microsoft Excel (Microsoft, Redmon, Washington, USA). The codebook is shown in online supplemental file 3).

## Patient and public involvement

The PPI group supported the design of the study, helping to identify priorities of inquiry.

## Findings

A purposive sample of 18 participants was recruited from physiotherapy and orthopaedic waiting and clinic lists at the University Hospitals of Derby and Burton NHS Foundation Trust. Some participants recruited from the orthopaedic waiting list received rehabilitation (pre and/or post) at another site. The sites at which they received rehabilitation varied across the Midlands and the detail regarding specific hospital departments was not collected. The study was discussed with 26 potential participants, four declined to participate and four did not respond after the initial discussion. Participant characteristics are shown in table 1.

Interview length was 25–51 min (mean: 38 min). Participants ranged from 18 to 45 years of age (median: 29 years), three identified as female (16.7%), with the remainder identifying as male (83.3%). Ethnic origin of participants was predominantly white (n=14, of which one participant also described themselves as Lithuanian), followed by Indian (n=2), British Asian (n=1) and Pakistani (n=1). 10 participants were awaiting ACLR, six were 3 months postsurgery and two were 1 year postsurgery. Injuries were sustained predominantly during a sporting activity (n=12), with the remainder occurring from a slip (n=2), landing from a jump (n=2), a motorcycle incident (n=1) and road traffic collision (n=1). All participants engaged in more than one activity type prior to injury, with 22 different types reported. Football was the most common activity (n=8), followed by attending the gym for muscle strength training alone (n=7) or cardiovascular and muscle strength training (n=5), swimming (n=4), rugby (n=3), running (n=3) and cricket (n=3). Other activity types are shown in table 2. The type of physical activity participants were engaged in at the time of the interview spanned a smaller variety, with muscle strength training the most commonly reported activity (n=7), followed by walking (n=5) and cardiovascular and muscle strength training at a gym (n=5). Activity types are shown in table 2. 12 participants engaged in prehabilitation (varied treatment length, type and frequency) at different sites across the midlands. The average number of days participants where physically active prior to injury was 4.5 days compared with 3.4 days at the time of the interview.

Five themes were identified from the interview data.

### Theme 1: injury experience, impact and support

Half of the participants reported an intuitive response to injuring their knee. Explaining an instinctive sense of its seriousness, reporting:

“I think immediately I sort of knew what I had done, even though I had never done it before” (P8)

Following injury, the route to diagnosis varied. Some described a seamless pathway where a direct referral was made from the emergency department (ED) to an acute knee clinic for specialist assessment and MRI. Others were advised to self-manage for an arbitrary period (typically 12 weeks) before seeking advice from a general practitioner (GP) or returning to ED if symptoms remained. Once diagnosed, several participants described this to be a difficult and distressing time. Beliefs about the injury and recovery were typically negative, with a consistent thought that surgery was essential. A lack of knowledge about the injury and its severity contributed to worry and catastrophising thoughts.

“During that early stage, I was panicking about what is happening … I was just assuming that my life is gone” (P14)

The burden of ACL injury impacted on self and self-identity, family, social and working life. The injury was described as having a profound impact on physical activity, with many concerned about causing further damage to their knee.

“I had to wait two years from when I found out it was an ACL rupture to trying to get surgery. …. I couldn’t do football. It was dangerous. Quit gym. I couldn’t go gym. …. I gained weight.” (P17)

Many described having to alter their working duties, finding alternative work or to be on long-term sick leave due to their ACL rupture. This resulted in social and financial loss. Several participants described physical and mental challenges of the injury, such as difficulty accepting changes to their body image. Participants reflected on feeling disabled and described several symptoms of depression.

“I have struggled really, really sort of deeply to the point where I didn’t want to go to work, didn’t want to get out of bed” (P6)

Existing personal support networks were valued, and support from those with prior experience of the injury seemed particularly important. There was some sense that healthcare professionals failed to fully understand the patient experience and therefore could not offer holistic care. It was also felt that mental well-being was not addressed by clinicians.

“When I went to the GP,… physio and the surgical consultant, there was no questions mentally there. … That’s something that I feel should be addressed. Because significant injuries like this are life changing and anything that’s life changing is very mentally draining” (P7)

### Theme 2: navigating the treatment pathway

Participants accounted for several challenges with the patient pathway, describing it as prescriptive and impersonal, like a ‘process’ (P2 and P3) where ‘you’re just going through the motions’ (P3). This led to participants feeling burdensome to clinicians and the healthcare system. Participants further reported the inconvenience of limited notice for appointments and surgery dates, in addition to frustration with delays and waiting times across the pathway. Participants described feeling lonely and undervalued due to clinician’s busyness and restricted appointment times.

There were multiple accounts of disjointed interactions that caused frustration and a lack of confidence in healthcare professionals, with some feeling as though they were responsible for coordinating all those involved in their care.

“I still don’t think there is that communication if I am honest … everything is on a database … even the physio … the first two or three times he kept going, ‘remind me what you have done’ … Obviously there has been no communication with the surgeon” (P8)

While some valued being an active participant in the communication loop, others felt this was disorganised and were frustrated with repeating their story. Those happy to feedback on previous conversations to the next healthcare professional felt this contributed to a sense of control over their care, which supported decision-making processes.

The primary decision discussed among participants was whether to proceed with ACLR. This decision-making process was described in three ways by participants, who (1) were not presented with an opportunity to partake in decision-making, that is, the decision was made for them (2) attempted to avoid decision-making in fear of feeling responsible should an incorrect decision be made and (3) did not feel they were presented with a decision as it was described to them that surgery was necessary to enable a return to physical activity. Several participants reflected on the lack of support with decision-making, which resulted in the feelings of helplessness and caused decision paralysis.

“I did not get a clear recommendation from the specialist on whether I should go for surgery, they gave me the personal choice. As a non-medical background person, I got the information from the internet and I am unable to make the decision. So, from my point of view, I was not able to make the decisions because I don’t know the severeness of the problem that I have.” (P14)

Unsurprisingly, many participants referenced COVID-19 to justify the shortfalls in their care. It was the predominant reason referred to for the delay in surgery, with participants offering some leniency because of this. Participants also reflected on the priority of their injury in comparison to others requiring medical attention during the pandemic. Understanding capacity within the hospital was typically measured against reports in the media, with participants starting to avoid attending the hospital when, perhaps, prior to COVID-19 they may have presented sooner.

### Theme 3: sense making in the preoperative period

Despite all participants being on the waiting list for or having had ACLR, some still recalled questioning its necessity during the preoperative period.

“I feel like I’ve just been on a pendulum – I want it done, no I don’t want it done, I want it done, no I don’t want it done, I want it done – and I’m still a bit like that, and a bit apprehensive” (P4)

**Figure 2:**
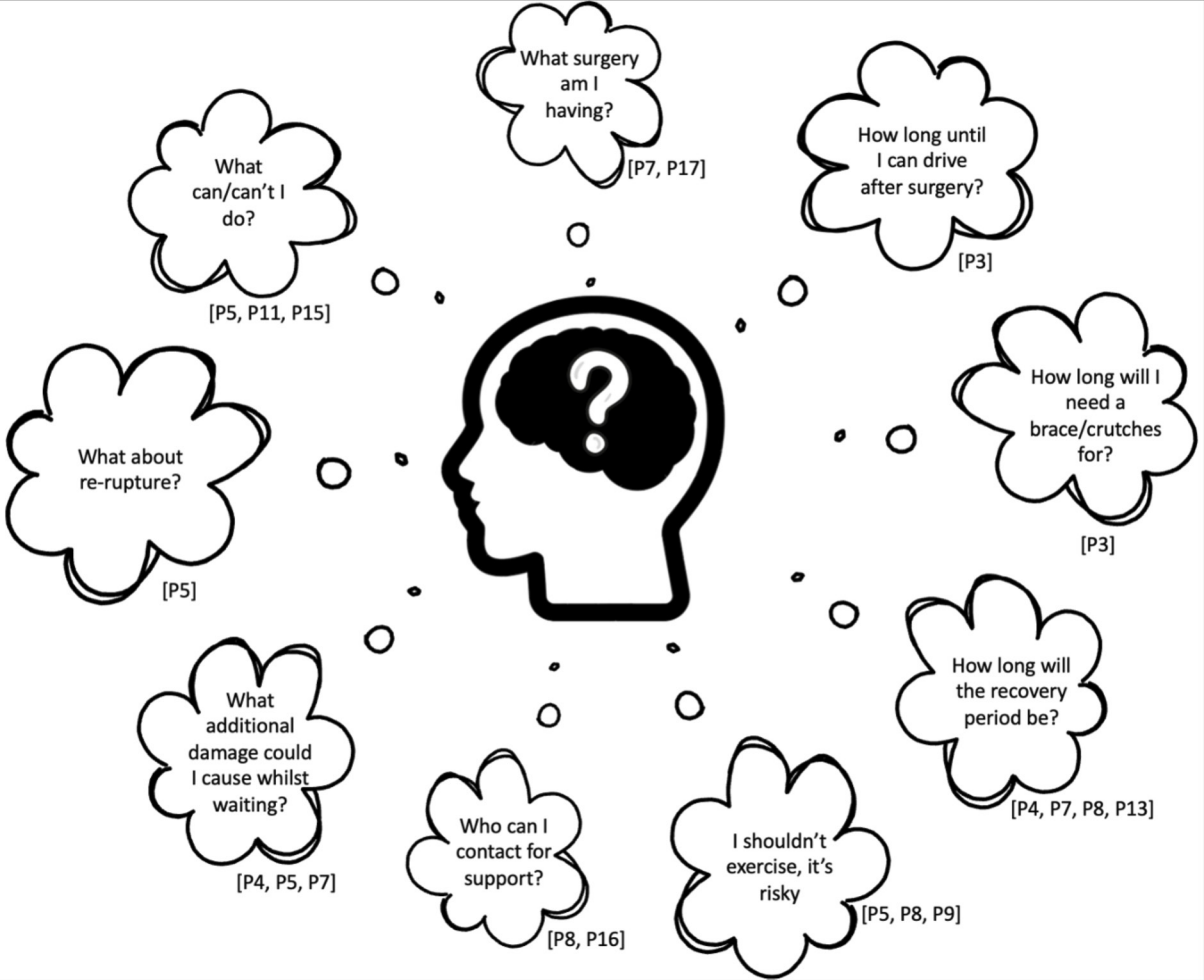
Concerns and questions raised during the preoperative period (supporting quotes are shown in online supplemental file 4).

Participants described feeling unsupported while awaiting surgery with several concerns and unanswered questions. Typically, these were related to knowing what to do while awaiting surgery, the surgical procedure itself or the postoperative recovery period. Examples are shown in figure 2 and online supplemental file 4).

Participants described lacking confidence during the preoperative period in (1) their decision-making, (2) ability to exercise, (3) understanding their identity as they adjust to their new lifestyle with their injury and (4) healthcare professionals/hospital procedures running as expected, that is, the concern of being missed off the waiting list.

Support through prehabilitation was valued highly among the participants, regardless of whether they had received it. It was perceived to offer an advantage to recovery, postoperative rehabilitation and support a faster RTS. Participants also felt it supported psychological well-being and increased their knowledge of the injury and its management. Where participants engaged in exercise with physiotherapy guidance it was described to develop confidence and offer reassurance that postsurgery exercise was achievable and may perhaps be easier. Prehabilitation’s utility in decision-making regarding surgery was also discussed. One participant explained to have only felt comfortable proceeding with surgery after exhausting their potential during prehabilitation.

“More a decision tool really, so, yes, I tried it and tried to push my boundaries with it but didn’t get where I want to be so, yes, it was more a decision tool for me.” (P2)

One participant felt they had nothing to lose by engaging in rehabilitation prior to surgery, describing desperation of trying anything to improve their knee function. However, there were concerns among some that prehabilitation lacked specificity, with participants describing disappointment when prescribed generic exercises. Where this was the case, participants struggled to understand the value of prehabilitation in their journey.

“I thought that it would be a little bit more personalised and there would be more of a road to recovery really … I just felt it (prehabilitation) was very generic” (P10)

Many participants wanted support transitioning from an unrestricted active lifestyle to managing the consequences of their knee injury, however, most felt that this need was or would be unmet by NHS services. Participants viewed prehabilitation as safe and valued the security of direction provided by a physiotherapist. Where prehabilitation was not offered, many described concerns about engaging in physical activity due to fear of their knee giving way, worry of causing further damage or experiencing pain and/or swelling.

### Theme 4: uncertainty, expectations and reality of the postsurgical period

Those at postsurgical time points reflected on the busyness of the hospital environment and delays experienced while being an inpatient. One participant accounted for feeling overwhelmed, rushed and confused due to the limited amount of time spent with medical staff on the ward.

“It is really quite confusing and overwhelming, you never really quite know what the plan is.” (P15)

The early postoperative period (≤3 weeks) was described as a particularly difficult time, due to unexpectedly high levels of pain, challenges caused by a lack of mobility, struggles maintaining morale and motivation and difficulties with managing thoughts/feelings independently.

“It’s about two weeks [before you] see anybody about it. So having that period where you’re expected to crack on with the exercises, in a lot of pain … and all you’re left is a booklet to read.” (P1)

Participants further described battling anxiety in the first 3 months postsurgery, concerned about the progress they were making, levels of pain and contemplating the success of surgery. Participants reflected on the intensity of postoperative physiotherapy and the challenge of balancing this against work and social commitments. Similar to preoperative rehabilitation, participants valued personalised care with respect to rehabilitation content and consultation medium.

There was a range of expectations regarding the return to work following surgery. Typically, these expectations were not addressed by healthcare professionals and were developed by the participant with little to no support. This is shown in table 4 with supporting quotes.

**Table 4:** Return to work expectations

Return to work expectations	Supporting quote
Following day after surgery	“Yes I’ll have to [work from home immediately], I’m self-employed. I’ve got people who work for me but most of the money comes from me doing, me doing stuff so I have no choice.” (P3)
Few days	I did tell work that I was going for surgery. And I told them, “Oh I just need like … three, four days to recover because it’s the weekend and then I’ll be back at work on Monday.” (P17)
6 weeks–3 months	“I think realistically I’ll be back to work in six weeks to three months” (P2)
2 years	“in my head I am just kind of going well I am going to be out for 2 years, I am not going to be able to work … I am just going to the worst possible scenario” (P7)
80% better	“I’ll just wait until it’s better, if it works then about 80% alright, then I’ll try it, but there’s no point in rushing it, is there, in case you damage it even more” (P13)

Expectations of returning to physical activity also formed a spectrum of assumptions with respect to time and ability to return. Time to return ranged from participants’ wishes for ‘as soon as possible’ through to questioning whether they wished to return at all. There was a range of expectations between the 6- and 12-month mark, with some feeling the time to return was based on their commitment to rehabilitation whereas others felt a measurement against time was the best indicator. When considering a return to physical activity, some questioned their confidence to return while others considered their ability to cope with and the impact of a subsequent ACL injury to their work and social life. Others were adamant to return with their confidence of being able to do so stemming from advice given by a healthcare professional:

“I was always set on it because the success rate that Mr. X gave was very high, like 95 plus to get my pre-injury levels. And I do believe that as well” (P17)

The ability at which participants felt they would be able to return also varied from not returning at all, accepting they would only be able to return at a subinjury level through to expecting a full return to preinjury level.

### Theme 5: Balancing resources, advice & opinions

There was a common narrative that resources were contradictory and difficult to navigate. Several participants stated mistrust with the internet and avoided searching their condition, in anticipation of inaccurate information that would conclude catastrophising outcomes.

“You know what Google is like, you look at knee surgeries and it turns out you are dying of something with the lungs, you know, don’t trust the internet.” (P7)

Many referenced the NHS website, although views of this resource and its reputability were conflicting. Participants described difficulty with their mental well-being and feeling “in the dark” (P7) due to absent information regarding surgery, how to prepare for it and what to expect postreconstruction. Information regarding timelines was another common frustration. In the absence of support presurgery, participants considered several hypothetical scenarios of how surgery and the subsequent recovery may impact their lives.

“Could my recovery time be six months plus or even longer, which if so, I could lose my job over that. I could really be up the creek … because I have got a child, a ten-month-old now … and a mortgage and my partner is still a full time mum, so we are relying purely on my income. Not knowing what’s happening with the surgery is really putting my stomach in my throat with regards to the future of myself and my family” (P7)

Advice regarding presurgery preparation was mixed. Some participants recalled the surgical team recommending physiotherapy and strengthening exercises while others were advised that this was not necessary and were instead advised against types of activity, for example, road running and swimming. Advice regarding surgery was unsurprisingly mixed, given the disparity in evidence. Where differing opinions and advice were offered, participants explained feeling confused, as they battled with deciding who to believe and trust.

“Well I don’t know who to believe now, do I believe the senior physio or do I believe the orthopaedic consultant?” (P4)

Some tackled this by only seeking advice from those they deemed reliable (predominantly surgeons and/or physiotherapists), which helped to keep the number of opinions they received low. Others gave descriptions of seeking confirmation bias of either their beliefs or beliefs of healthcare professionals they felt they could trust.

An important reflection from this study is the impact of culture, with a unique viewpoint offered by two participants who compared UK NHS treatment to that offered in Lithuania and India. One participant described advice from a surgeon in Lithuania that optimal treatment was surgical intervention 6 weeks postinjury:

“So, in six weeks, that was his words, when the swelling goes down and starts healing then you get your surgery.” (P12)

The second participant described being recommended ACLR by a surgeon when visiting India in addition to the use of complementary medicine (such as herbal oils) to support healing, pain management and muscle strength by a physiotherapist. They reflected on the conflicting advice from clinicians in their home country compared with that offered in the UK. These reflections are an important consideration in the treatment of patients who may come from cultures with different health beliefs and/or have access to care in a different health system with alternative views and practices.

## Discussion

This study is the first qualitative exploration of patient experiences specific to the NHS ACLR pathway.

A key finding from this empirical work is the difficulty experienced by participants when navigating the NHS treatment pathway. This was evident at pivotal points from initial injury management to surgery. Challenges were highlighted with (1) referrals to appropriate clinicians to support timely diagnosis and appropriate management, (2) coping independently in the preoperative period, (3) communicating with healthcare professionals (eg, liaising with multiple professions, gaining support with concerns/questions/updates on treatment time-frames), (4) making decisions about injury management and (5) patient-centred, personalised care.

Patient experience has been identified to be positively associated with treatment outcomes and patient safety across a range of conditions, settings and patient groups.^19^ A common narrative in this study was the lack of consistent information and reliable resources regarding ACL treatment and outcomes; this has previously been identified to affect patient experience outcomes.^20^ A number of participants in this study referenced the NHS website. However, ACL information available on the NHS website is not consistent with the evidence base. Accessed in August 2023, the NHS website states: ‘ACL surgery fully restores the functioning of the knee in more than 80% of cases’.^21^ There is no reference supporting this claim and ‘functioning of the knee’ is unclear and open to interpretation. It further states that recovery following surgery ‘usually takes around 6 months, but it could be up to a year before you’re able to return to full training for your sport’. This does not match the literature, which no longer recommends a return based on time alone and it is commonly acknowledged that a return may take up to 2 years.^22^ Surplus amount of information has further been identified to contribute to poor patient satisfaction and the importance of supporting consultations with written patient information has previously been identified as important.^20 23^ This empirical work highlights this gap in clinical care and the need for consistent information for the ACL population that is readable and viewed to be reliable. It may further benefit patient understanding and thus satisfaction, for clinicians to directly address patients’ internet findings to ensure they are correctly informed. This would further support shared decision-making, of which there was limited evidence in this study.

Finally, a key element highlighted by participants was the importance of tailored, patient-centred care. This was in reference to treatment discussions, provision of written information and rehabilitation programmes. Descriptions of generic exercise prescription by participants perhaps mirror the lack of consensus in the literature for this stage of treatment.^4 24^ Inconsistencies in care were evident in this study, despite all participants receiving treatment within the same UK region, predominantly at one hospital. With widespread financial restrictions across NHS services, understanding optimum treatment is important to inform clinicians and financial stakeholders as services that lack clear guidance are likely to be cost-inefficient.

### Clinical implications

There are a number of messages important for clinical practice arising from this research. First, failure to recognise a suspected ACL rupture and referral onto an appropriate management pathway remains an issue, with several participants failing to be referred for specialist assessment after ED attendance. Getting it right first time is a current national initiative in the UK.^25^ Suboptimal management results in delays to diagnosis and treatment in addition to increased healthcare and economic costs.^26^ A 2015 NHS study reported a reduction in days to diagnosis and treatment of ACL ruptures following implementation of an acute knee clinic;^27^ demonstrating the success in getting it right first time.

Second, greater attention needs to be paid to decision-making regarding injury management. This was particularly evident for those questioning the necessity of surgery during the preoperative period. Shared decision-making has been shown to improve patients’ knowledge, help patients and clinicians to understand preferences for treatment, reduce decisional conflict, help to clarify and set realistic expectations, and increase patients’ involvement in their care.^28^ It is outlined by the National Institute for Health and Care Excellence (NICE) as a process by which patients and clinicians work collaboratively to determine investigations, management plans and support needed based on individual preferences and relevant evidence.^29^ Findings from this study demonstrated variation in patient involvement in decision-making conversations. Motivation to be involved in decision-making is multifaceted, influenced by individual preferences, level of risk, fear of a negative outcome, perceived importance of the decision and cultural, social and economic factors.^30^,^32^ In the absence of a tool specific to the ACL population, we suggest that clinicians seek to understand patient’s treatment preferences, values and beliefs, their preferences to be involved in the decision and factors that may affect this and use up-to-date evidence to educate patients on their options to support informed, shared decision-making. Further, it was felt that mental well-being was not addressed by clinicians, despite musculoskeletal and orthopaedic injuries being commonly linked to poorer mental health outcomes.^33^ This could be considered by clinicians to ensure appropriate support, and signposting is provided to patients to manage their condition and make decisions about their care.

Third, signposting patients towards reliable information should be considered to support face-to-face discussions and patient education conversations. Educating patients on the specificities of their condition and how this differs from ‘generic’ advice is another important reflection of this study. Encouraging patients to present contradictory advice/information may also support patient understanding of their condition and consolidate their ability to make clear, informed decisions.

It is important to acknowledge that participants in this study were recruited from one hospital Trust but received rehabilitation at a range of different sites within the Midlands. While data on specific sites were not collected, the experiences discussed may not represent those of other areas of the UK.

### Research implications

This research highlights the perceived benefit of preoperative rehabilitation and support among ACL patients. Current evidence supporting preoperative care is limited^4 24^ and so further exploration of this phase of treatment is warranted. The development and evaluation of preoperative interventions that address patients’ understanding of their condition, decision-making ability and optimal physical and mental preparation for surgery is needed. Understanding what warrants optimal preparation also needs further consideration, with input from both patients and clinicians. The evaluation of these interventions on patient-reported outcomes, clinical measures and cost-effectiveness would further support the delivery of optimal and cost-effective treatment pathways.

The absence of a decision support tool for this patient group has been identified. A 2021 systematic review highlighted that shared decision-making implementation research in hospital settings is an emerging field and an important area for further work.^34^ Implementation factors are an important consideration to ensure decision support tools are developed with their context of use in mind. We have discussed the potential benefits to patient and clinical outcomes of implementing such a tool. Further work to develop and evaluate this would be a novel area for future exploration.

This research also recognised the importance of high-quality patient information resources which are currently lacking for this patient population. Future codevelopment work to develop these resources may also be beneficial.

### Strengths, limitations and reflexive considerations

Participants were given the choice of a face-to-face or virtual interview. During study design, PPI members felt this was important to offer flexibility to participate in addition to considering personal preferences. In-person interviews were carried out in a hospital setting. While private rooms were arranged and participants were reassured of anonymity, participants may have felt less comfortable recounting negative experiences when physically present in front of the researcher who also identifies as a member of the clinical team.

In addition, the same topic guide was used across interviews and HC was responsible for guiding questioning and probing topics. A reflexive journal was kept to account for experiences and initial responses after each interview to raise awareness towards the channelling of questions. These points were considered during data analysis and mitigated by collaboration with the research team, trial management group and trial steering committee which includes patients and stakeholders.

Another important factor to consider, impacting patient experience on the surgical pathway, is the timing of this research study. Interviews were carried out two and a half years after the COVID-19 pandemic reached the UK. COVID-19 had a profound impact on NHS services with many face-to-face procedures delayed and the rapid implementation of virtual consultations.^35^ Although the NHS recovery plan for managing the backlog of elective procedures was rolled out in February 2022,^36^ service demand and capacity remained unevenly balanced. Unsurprisingly, several participants attributed shortfalls in their care to COVID-19.

Finally, the study population was treated within hospitals in one region of the UK (Midlands). We did not collect data to detail all hospital departments in which patients received rehabilitation treatment. While it is not the aim of qualitative research to be generalisable, these findings may not represent the experiences of those treated in other UK regions and outside of an NHS setting. However, a purposive technique was employed, which allowed sampling to be responsive to emerging data. Further, there was an inconsistent number of participants at the three identified time points for the study. There were a greater number of participants at the preoperative time point which is likely to have resulted in a greater richness of data regarding the preoperative pathway than that of the postoperative pathway (particularly at the 1-year time point, where only two participants were interviewed).

## Conclusion

This study has illuminated patient experiences of the NHS ACL surgical treatment pathway, novel to the ACL evidence base.

It highlights the gaps in patients’ support and the magnitude of issues patients face when navigating the NHS system, communicating with clinicians, making decisions about surgery and managing conflicting sources of healthcare advice. These issues have not previously been recognised in the literature.

## supplementary material

10.1136/bmjopen-2023-079468 online supplemental file 1

10.1136/bmjopen-2023-079468 online supplemental file 2

10.1136/bmjopen-2023-079468 online supplemental file 3

10.1136/bmjopen-2023-079468 online supplemental file 4

## Data Availability

All data relevant to the study are included in the article or uploaded as supplementary information.
