# Supplementary material for: ‘Going through the motions’; a rich account of the complexity of the anterior cruciate ligament reconstruction pathway, a UK qualitative study
Source: BMJ Open. 2024 Sep 17;14(9):e079468. doi: 10.1136/bmjopen-2023-079468 (PMC11409353; doi:10.1136/bmjopen-2023-079468)
Supplement: online supplemental file 2 [file bmjopen-14-9-s002.pdf]

The University of Nottingham
UNITED KINGDOM · CHINA · MALAYSIA

NHS
University Hospitals of Derby and Burton
NHS Foundation Trust

Supplementary File 2

Interview Topic Guide – Preoperative / 3 Month / 1 Year Postoperative Interview

(delete as appropriate for interview time point)

Version 1.0 – 31/05/2022

Introduction and Background

- Thank you for agreeing to take part in the study to discuss you experiences
- Tell me about your journey so far

Possible prompts:

- Timeline of injury and initial management
- Waiting times to surgery and support given in that time
- Knowledge of [include as appropriate – planned] surgery and postoperative rehabilitation

Topic 1: Expectations of treatment and returning to physical activity

Possible prompts:

- Expectations of treatment, returning to physical activity and work after surgery
- Who have expectations been set/influenced by
- Have they changed since diagnosis
- Expectation versus reality of current ability

Topic 2: Views and involvement in prehabilitation

Possible prompts:

- Engagement in physical activity [delete as appropriate - since injury / prior to surgery]
- Specific referral for prehabilitation
- Description of rehabilitation intervention e.g. advice, exercise
- Views of prehabilitation and what it should/should not involve
- [Delete as appropriate - Engagement in postoperative rehabilitation]

Topic 3: Healthcare advice

Possible prompts:

- What advice has been given
- Who has given the advice e.g. healthcare professional, friend/family member, internet
- Conflicting advice

Close

- Is there anything you feel could have been managed differently by your healthcare providers e.g. surgeon, GP, physio
- Anything further you would like to mention/discuss
- Thank you for taking the time to discuss your experience
