# Supplementary material for: ‘Going through the motions’; a rich account of the complexity of the anterior cruciate ligament reconstruction pathway, a UK qualitative study
Source: BMJ Open. 2024 Sep 17;14(9):e079468. doi: 10.1136/bmjopen-2023-079468 (PMC11409353; doi:10.1136/bmjopen-2023-079468)
Supplement: online supplemental file 3 [file bmjopen-14-9-s003.pdf]

Supplementary File 3 – Codebook

**Theme 1**: Injury experience, impact and support	**Theme 2**: Navigating the treatment pathway	**Theme 3**: Sense making in the preoperative period	**Theme 4**: Uncertainty, expectations and reality of the post-surgical period	**Theme 5**: Balancing resources, advice & opinions

No.	Code	Definition	Coding Coverage	Exemplar Quote

Theme 1: Injury experience, impact and support

1.01	Impact of ACL injury	Descriptions of the impact of the ACL injury to the participant	57 references across 14 interviews	*"I have struggled really, really sort of deeply to the point where I didn't want to go to work, didn't want to get out of bed. Obviously you are young and when you are so used to having a certain lifestyle"*
1.02	Blame for injury	Descriptions of blame for sustaining the ACL injury	7 references across 6 interviews	*"I was stationary at a set of traffic lights when a car doing nearly forty miles an hour crashed into the back of me. It was an elderly gentleman who had just come from the hospital, so he was still under medication when he sort of, he bumped me ... I had been playing football for years and never really hurt myself so maybe it was something that was due to come."*
1.03	Actions of the patient re mental health	Actions taken by the participant regarding their mental health	15 references across 11 interviews	*"Being honest I went and got a dog. I went and got a greyhound which needs walking. So I take him out for walks so he can pull me a little bit and I take the children out for walks and stuff. So I have been a little bit more family devoted in respect that I don't really spend that much time on my own anymore, it's always me with somebody else. But I think that was a comfort thing for myself because when I was able to take the dog and take my son out, or take my dog and take my kids out, or just me and my partner and my dog would go for a walk, it was like a de-stress. It was like shifting my focus onto something different""*

1.04	Patient thoughts re mental health support	Descriptions of the participants thoughts regarding the mental health support available	19 references across 7 interviews	*"I think the support would have been definitely helpful and it might have made some changes really to how I felt but that's just how it is I suppose"*
1.05	Support from those with similar experience	Descriptions of support received or wanted by the participant from those with similar experiences e.g. an ACL injury	10 references across 5 interviews	*"I think you can only get so much information from doctors, nurses, and physios and stuff, if they have actually gone through it themselves. You can get a very different picture from someone who has had the surgery and gone through all of the rehab process and how they found it and the setbacks and things like that."*
1.06	Opportunities to seek help	Descriptions of the opportunities for the participant to seek help	6 references across 6 interviews	*"unfortunately I haven't really had a chance to speak to anyone with regards to any questions I have got"*
1.07	Knowledge of injury	Descriptions of the participants knowledge of their injury	14 references across 9 interviews	*"All I have heard from the doctors is that kind of throw away comment of oh that's quite a bad ACL tear isn't it?"*
1.08	Knowledge of ortho interactions pre surgery	Descriptions of the participants knowledge of orthopaedic interactions e.g. appointments prior to surgery	4 references across 3 interviews	*"I just don't know what to expect from now, what kind of like procedure there is or progression there is from now. I am just kind of waiting to hear anything"*
1.09	Knowledge of surgery & postoperative rehabilitation	Descriptions of the participants knowledge of surgery and postoperative rehabilitation	54 references across 14 interviews	*"'ve definitely got surgery in six weeks, I run a company, I've got staff members. I know I'm going to have to be at home. Do I know how long I can't drive for? Do I know how long I'll be in a leg brace? You know how long on crutches for? I know absolutely nothing"*
1.10	Thoughts about recovery from ACL tear	Descriptions of the participants thoughts about recovery following an ACL tear	27 references across 15 interviews	*"Not able to swim and not able to muck about with the kids … I don't think I'll get to that point until I've had a repair"*

1.11	Thoughts about how other's might have dealt with their situation	Accounts and reflections of how the participant feels other may deal/cope in their situation	1 reference across 1 interview	*"other people they do struggle don't they? ... it would be hard and some people wouldn't just accept it because they feel like a victim and probably rightly so but that's the, depends how you see things don't it? Everyone's different"*
1.12	Reactions to negative stories of ACLR	Participants reactions to hearing negative stories of others treatment of ACLR	11 references across 9 interviews	*"any stories of people becoming worse off after the surgery kind of I dismissed"*
1.13	Outlook on life	Participants reflections/descriptions of their outlook on life	9 references across 7 interviews	*"I worked in sales for seven years of me life. It's dead cut throat etc, etc. I set up me own company at 26, got people working for me. If you're not like, you can't beat around the bush so that's my outlook on life. You get in what you get out. Put in what you get out of it"*
1.14	Ability to cope	Descriptions of the participants thoughts about their ability to cope with adversity/their ACL injury	6 references across 3 interviews	*"I just prefer not to know and go in with an open mind, do everything they have asked me to do. If I get through it I get through it. If I don't then that will be something I have to reconsider with regards to my mental health"*
1.15	Importance of physical activity	Descriptions of the important of physical activity to the participant	11 references across 8 interviews	*"looking at the importance of football to myself I think it was like I use it as kind of like a way out psychologically as well as keeping fit so and I've played since I was quite young so around seven so it's quite engrained in me"*
1.16	Actions after initial injury	Descriptions of the participants actions after sustaining their initial knee injury	24 references across 17 interviews	*"I didn't go to the- Because we were out with friends, and I was out with my daughter and you sort of try and hobble along and cope. And then we went for dinner and I stayed out and then when I tried to get up after the meal I was like, I literally can't walk, really, very well. So then it was like, I think maybe I do need to go to A&E"*
1.17	Actions whilst awaiting/pre-diagnosis	Descriptions of the participants actions prior to diagnosis of an ACL rupture	10 references across 7 interviews	*"In the meantime [whilst awaiting a diagnosis] I did start kind of going to the gym and trying to strengthen my leg and get back to normal kind of walking as much as I was able to"*

1.18	Thoughts about the initial injury pre-diagnosis	Participants thoughts about their initial injury prior to diagnosis of an ACL rupture	25 references across 15 interviews	*"I've just gone down to the floor and I knew what I'd done because there was that sound and pain and loose leg, and it just started pumping up quite quickly"*
1.19	Thoughts about the injury post-diagnosis	Participants thoughts about their injury post diagnosis	17 references across 9 interviews	*"I was panicking, I was just assuming that my life is gone, trust me. I've gone to that level because I don't know what is the implication of ACL. So, you are physically fit and you are a normal person and all of a sudden, someone is saying you have got an ACL problem"*
1.20	Experiences of diagnosis	Descriptions of the participants experience of their ACL rupture diagnosis	44 references across 17 interviews	*"So it was just oh we have done the X-ray yes, no there is nothing wrong with you, you have just got a bit of swelling. No there is something definitely wrong with me. Oh what we will do is we will do an ultrasound, oh yes look listen there is still definitely nothing wrong with you. I am telling you there is. I know my knee. I know my body, something is not right. Okay, well we have done everything I just think it's the after effects of the accident. Okay, so what I will do is I will go to the doctor then every time I feel pain, which is obviously quite regular and then this is what happens.<br>They referred me. The specialist chased it up and then I got the correct diagnosis."*
1.21	Experiences of (potentially) missed diagnosis	Descriptions of the participants experience of encounters with healthcare after their knee injury where an ACL rupture diagnosis was not made	22 references across 9 interviews	*"I was annoyed that it was missed but then again honestly, it's just - Well I went to A and E and they - Well they did the test for the ACL but I guess my knee was swollen at that point so they couldn't really see it. But I was annoyed when it was missed because after that I thought it was all fine that I could do what I want and then when it collapsed on me in the snow, I was in a lot of pain it took what from that point a month and 1/2 until I got diagnosed with it"*
1.22	Second injury	Descriptions of a second knee injury	12 references across 7 interviews	*"I left it and went back to playing football. In January, I think it was January the 6th or 7th, probably no further distance from about a metre away, someone just passed me the ball, I opened up my leg, or my foot to a right angle again and it just popped and exploded and within about a minute, it was the size of a football. And at that point, I knew that I had actually done something quite bad to it"*

1.23	Actions of the patient after reinjury	Descriptions of a the participants actions after their second knee injury	24 references across 17 interviews	*"I was throwing snow balls at my sister and then it just completely gave way and that's when I got in touch with my GP and they referred me for an MRI"*
1.24	Comparing previous experiences of ACL injury/surgery with current experience	Descriptions/reflections of previous ACL injury experiences and ACLR	9 references across 1 interview	*"This time I'm a bit surprised how long it takes me to build myself up, but now, it makes sense, because the last time, I had that pre-surgery physio and that could be the reason, exactly that's the reason why. Last time, it didn't take as long as I'm doing it now, even though it is a very similar surgery that has been done"*

Theme 2: Navigating the treatment pathway

2.01	Making decisions	Descriptions of the patients decision making processes and thoughts about decision making	54 references across 16 interviews	"I want absolving of any responsibility. I just need to, because I've never really had to have any surgery before, either. So it's, you know, I don't want it if I don't have to have it, but if I do need to have it, then I will have it. Do you know what I mean, so I want somebody to tell me whether I need to have it."
2.02	Factors involved affecting the participant/their choices	Descriptions of factors considered by participants that affected them/their choices	52 references across 14 interviews	"Probably a slight influence would be family, because my father went through the exact same injury"
2.03	Suggestions or references for pathway improvements	Suggestions from participants on improvements that could be made to the ACLR pathway	84 references across 14 interviews	"[I would have liked] having a bit of physiotherapy input before the surgery as well, just to help prepare myself for what would happen afterwards, but also just to improve my level of function beforehand as well."
2.04	Experiences/thoughts of the pathway	Participants descriptions of their experiences and thoughts of the ACLR pathway	33 references across 12 interviews	"I was frustrated that when I went to A&E to get it checked they just, I don't want to say palmed me off as such but they just said, "Oh it's bruised. It will heal within a few weeks." That kind of annoyed me when later on I found out that I've got a stability issue three months down the line and then the GP had to get involved to send me for an MRI."

2.05	Elements valued by participants	Participants descriptions of elements/factors they valued across their care	53 references across 11 interviews	"the surgeon was fantastic. It was quite a younger gentleman, I think he was like mid-thirties. He was quite a sporty person himself so he was dead relatable, he was very friendly. And he was basically saying look I can't sort of say to yourself to go for something or not, but what I am saying to you is if you did want to go and play sports again that this would be your option. However, please note that there is a risk that it will not work and that you could consider not still playing sports like you do now. So he was honest but like I said he was very friendly and understanding"
2.06	Interdisciplinary communication	Descriptions of participants perceptions/experiences of communication between healthcare professionals e.g. orthopaedics and physiotherapy	17 references across 7 interviews	"I will call up my GP and be like hey what's going on with this? And they will be like contact the orthopaedics and they will be like contact your GP and, you know, you are just going round in circles."
2.07	Communication between the participant and healthcare professionals	Descriptions of participants experiences of communicating with healthcare professionals	54 references across 15 interviews	"No they give you the pamphlet but they basically they don't go through it with you they just go here's some documents. You get handed it at the end of the thing and then you walk out. Do you know what I mean they don't run through it with you so it's just this is that, it's all just very here's some documents you might find useful, see you in a bit basically"
2.08	Communication during inpatient stay	Descriptions of participants inpatient experience	3 references across 3 interviews	"I think while you're on the ward as an inpatient it is in and out so you're being seen by the nurse, they pop out, the consultant out, physio out. So I don't think from a patient point of view it doesn't seem very connected in terms of how everyone is collaborating for yourself on the ward"
2.09	Participants' role in communication	Descriptions of the participants role in communication with healthcare professionals	14 references across 9 interviews	"I couldn't remember who I had to ring, so, I just left it to them to get in touch with me."

2.10	Points of contact	Participants descriptions of people they contacted/knew where available to contact for support	8 references across 5 interviews	"Like who do you go to? Do you go the surgeon, do I speak to the receptionist? Do you go to the physio who you have not seen in the last year? Do you go to the GP who you are not going to get seen because GPs are difficult to get hold of at the best of times, never mind if it's not an urgent case where you aren't going to drop dead the next day? So it's like where do you go? You are not being given any information. Do you go back to A&E? A&E is absolutely rammed at the minute with patients left, right and centre"
2.11	Advice/actions sought from GP	Descriptions of advice sought from or actions of the participants GP	25 references across 10 interviews	"I don't like to go to the hospital because I know there is a lot of people that need it more than myself, so I don't like to feel I am wasting any time. So I booked the earliest appointment at the doctors on Monday morning and then they sent me to the hospital"
2.12	Experience of GP interactions	Participants experiences of interactions with the GP	10 references across 6 interviews	"my previous GP unfortunately ... had never passed me on or they had not passed on the right information ... then I moved house, got a new doctors, a new GP earlier this year, which I then said right what's going on? I spoke to my new GP and they basically said I have no idea why you have not been contacted yet regarding this ... my new GP basically got the wheels in motion"
2.13	ED actions	Descriptions of actions taken by the emergency department after participants visited with their knee injury	21 references across 13 interviews	"I went to A&E, I think it was that evening. I think I went straight there pretty much and just explained what had happened and how the injury had taken part. They literally thought it was a meniscus tear because of where the pain was and they said come back in six weeks' time if you still have any pain, any difficulty with walking etcetera"
2.14	Experiences of orthopaedic interactions	Participants descriptions of interacting with orthopaedics	43 references across 14 interviews	"I first saw a consultant and he kind of didn't listen to my problems as much, so I decided to get a second opinion about it and then I felt I was listened to a bit more"
2.15	Thoughts about seeking private treatment/paying for healthcare	Participants thoughts about seeking private healthcare and/or paying for investigations and treatment	18 references across 6 interviews	"We did look at having it done privately but then that was like eight thousand pound. And then I have got a wedding to save for and it's like I don't think the wife is going to be too happy if I take eight grand out of the wedding budget"

2.16	Thoughts about the NHS	Participants thoughts about and reflections of the National Health Service	14 references across 8 interviews	"it's just a case of getting through with the NHS, getting through to the right person that you need to"
2.17	Thoughts about virtual interactions	Participants thoughts about virtual interactions/consultations/appointments	4 references across 2 interviews	"I'd have thought you would need to see somebody in person to see what was up with them"
2.18	Impact of COVID	Participants reflections of the impact of COVID on their ACL injury assessment, management and treatment/hospital care and procedures	26 references across 11 interviews	"because of Covid all the operations and everything got put on hold for like two years. So I had to wait two years from when I found out it was an ACL rupture to trying to get surgery basically"

Theme 3: Sense making in the preoperative period

3.01	Descriptions of preoperative physiotherapy	Participants descriptions of preoperative physiotherapy treatment	30 references across 13 interviews	"gave me an exercise and told me to practice them at home, they showed me how to do them there and just said practice these when you go home"
3.02	Experience of rehabilitation/physiotherapy first	Participants accounts of their experiences of undergoing rehabilitation, as recommended by their clinician, for the management of their ACL injury prior to being listed for surgery	3 references across 1 interview	"I waited for six months then after six months, I went to the physio rehabilitation centre, I did all my physios, and then they put me back to an orthopaedic specialist"

3.03	Experiences of preoperative physiotherapy	Participants descriptions of their experiences of undergoing preoperative physiotherapy	37 references across 11 interviews	"So I have been home with exercises and I never really got a follow up or anything like that. And still to this day I didn't hear anything from them until I started chasing it up again at the start of 2020"
3.04	Participants thoughts about engaging in physical activity pre-surgery	Participants thoughts about engaging physical activity with their ACL rupture, prior to ACLR	11 references across 7 interviews	"I thought I can't exercise anymore because anything I do will cause me pain or swollenness again, I will do more damage to it"
3.05	Participants thoughts about preoperative physiotherapy/prehabilitation	Participants thoughts about preoperative physiotherapy/prehabilitation +/- how it may have helped	77 references across 15 interviews	"For me, personally, doing the prehab has helped me continue with daily activities, so if I didn't do it, I probably would be a lot worse and I'm now in a better position to have surgery because my tissue's healing well and stuff."
3.06	Participants thoughts about what prehabilitation should contain	Participants thoughts as to what they think prehabilitation should include to support surgical intervention	18 references across 8 interviews	"probably early stage it's just focus on getting the swelling down, working on more stretching, just to gain that range of movement back, being patient with it, because you can't rush rehab, and then gradually just strengthening"
3.07	Justification for stopping preoperative physiotherapy advice/exercises	Descriptions of particpants reasoning/justification for discontinuing or going against advice and/or exercises given during the preoperative period	5 references across 4 interviews	"in the end I will be honest I left them [the exercises] because again I felt like I was getting no benefit and it was a bit, it was a bit tedious to be honest"
3.08	Other treatment/adjuncts	Descriptions of treatments and adjuncts other than exercise used by the participants during the preoperative period	9 references across 5 interviews	"I went and got a knee brace"

3.09	Participants expectations of the wait for surgery	Descriptions of the participants expectations of the wait for ACLR	10 references across 9 interviews	"obviously with Covid he did say to me that the wait was going to be quite a long time because of the situation, so I was prepared for that at the time"
3.10	Participant experiences of waiting for surgery	Descriptions of the participants experiences whilst awaiting ACLR	42 references across 14 interviews	"Maybe it's not that bad, maybe I'll wait, maybe it's not that long, maybe they'll call me how long will it be? You just sort of blindly expect the best"
3.11	Descriptions of thoughts/feelings pre surgery	Descriptions of the participants thoughts and feelings leading up to ACLR	36 references across 9 interviews	"I feel like I've just been on a pendulum - I want it done, no I don't want it done, I want it done, no I don't want it done, I want it done - and I'm still a little bit like that, and a bit apprehensive"
3.12	Actions of the participant whilst awaiting surgery	Descriptions of actions of the participants whilst awaiting ACLR	17 references across 8 interviews	"over time I was like, look I've had enough. I'm going for my walks in the Peak District and at that point I kind of assessed it myself. I was like, oh I can walk okay but I just have to be a bit careful not to do sudden movements. I just have to be mindful that my leg is not 100%."
3.13	Function during the preoperative period	Descriptions of the participants functional ability during the preoperative period	23 references across 10 interviews	"I can't do that—I can go swimming and that still but it hurts afterwards. And if I'm walking down the road, I used to do quite a bit of walking, if I took the dog for a walk, I wouldn't be able to walk the next day."
3.14	Description of current state - preop	Participants description of their current state during the preoperative period	26 references across 8 interviews	"'ve been tempted to try and play some sport, but it's not been great with it, so I've just left it and focused on more general rehab physical activity"
3.15	Thoughts about surgery	Participants thoughts about surgery	49 references across 18 interviews	"I don't really want to have surgery, but I don't want to be living my life saying, "No." To the kids."
3.16	Participants questions about surgery	Participants questions about surgery	9 references across 6 interviews	"it made me a bit nervous, to be fair, because I don't know what to expect, and I don't know what's going to happen, I'm a bit nervous. I've never had an operation before, never, so, I don't even know what to expect"
3.17	Thoughts during preoperative period	Participants thoughts during the preoperative period (not about surgery)	23 references across 10 interviews	"What's going on and what's happening? ... It's been ages since I've seen anybody. Since I went to the hospital, it must be over a year and a half"

3.18	Things missing from preoperative period	Descriptions of things participants felt were missing from the preoperative period	24 references across 13 interviews	"Well to explain what the surgery entailed and what your recovery's going to look like … I don't know, just a bit of reassurance really, you know. "

Theme 4: Uncertainty, expectations and reality of the post-surgical period

4.01	Experiences of surgery	Descriptions of participants personal experience of surgery	5 references across 3 interviews	"So I had a spinal block and I was actually watching it. It looked like fluffy clouds to me but the fluffy clouds was my ligament basically"
4.02	Experiences as an inpatient	Descriptions of participants experiences during their inpatient stay	14 references across 5 interviews	"I was here at 7 o'clock because I had to be, I couldn't eat, couldn't drink, and I didn't go into surgery until half 4 ... I did stay overnight as well, which I wasn't meant to, I was meant to come and wake up and go. I had to stay overnight, which wasn't ideal, and then I didn't get let out until I think about 5 o'clock the next day. So, in terms of how I was set up for how it would affect me moving forward, it was almost a bit of—You can't drive, use your crutches, we'll be in contact about physio and that was it and I thought, okay, see you later."
4.03	Experiences in the early postoperative period	Descriptions of participants experiences in the first few weeks post-surgery	23 references across 5 interviews	"I found it really, really difficult and frustrating just being in bed for that first week or so, and particularly the pain, I just wasn't expecting that that was quite difficult. Definitely, at certain points in those first couple of weeks, I questioned whether I should have had the surgery or not."
4.04	Experiences postoperatively	Descriptions of participants experiences following surgery	24 references across 5 interviews	"I think initially, it didn't feel too hard but then I think you get to a certain point where it does feel quite intense and trying to fit that around university and work and things like that is quite difficult as well"
4.05	Participants thoughts postoperatively	Descriptions of participants thoughts following surgery	6 references across 3 interviews	"I've got different feelings, a lot of feelings in my leg, so, that makes you think a lot like, why are these muscles not building back; why is it taking so long, is everything alright? So, you start to overthink everything now, I'm at that point now. Did they do a good surgery, do I need another, because I know there are so many unsuccessful ones and you do have to—Is that right? So, yes, overthinking definitely by this point."

4.06	Thoughts about postoperative progress	Descriptions of participants thoughts about their progression following surgery	11 references across 6 interviews	"As I said, it's frustrating now because we're talking—June, July, August, September, October, so, it is three months and I still can't bend fully without pain"
4.07	Thoughts about postoperative rehab	Descriptions of participants thoughts about postoperative rehabilitation	35 references across 12 interviews	"I feel like the stuff I have been given post-surgery has been more detailed and more explanation as to why we are doing this."
4.08	Expectations postoperation	Descriptions of participants expectations of the postoperative period	14 references across 10 interviews	"once I've had the surgery and the recovery process is complete, don't get me wrong there will still be that lingering doubt but it will be a lot less and then over time it will just be eliminated because you will establish confidence in your knee"
4.09	Expectations of RTW post surgery	Descriptions of participants expectations about returning to work following surgery	9 references across 8 interviews	"I expect to go back to work probably six weeks"
4.10	Expectations and thoughts about return to preinjury sport/physical activity	Accounts of participants expectations and thoughts about returning to their preinjury levels of sport or physical activity	49 references across 16 interviews	"I am between a rock and a hard place. I am thirty-five years old and I don't know, should I play rugby being a police officer? I would like to, to be honest, I never want to stop. But I also need to take into consideration the probability of injury"
4.11	Expectations vs reality	Participants descriptions and reflections of the reality of their situation versus their prior expectations	8 references across 3 interviews	"You don't necessarily think about the setbacks that you might have and how difficult you might find that. You just have this idea that you'll have the surgery, you might have a bit of pain for a few days and then gradually, you'll just keep making progress. Whereas in reality, you might have setbacks or your progress might level off for a while and you might not see much improvement and dealing with that is quite difficult"
4.12	Experiences of RTW postoperatively	Descriptions of participants experiences of returning to work following surgery	3 references across 3 interviews	"I was working the day after my surgery. Because I was working from home and I was like well I am just going to be sat here anyway."

4.13	Experience of return to impact	Descriptions of participants experiences of returning to impact activities following surgery	3 references across 1 interviews	"It felt very weird the first few times I did it, I remember when I started to jog, it was like I had completely forgotten how to do it and I was overthinking everything. And it's quite scary as well, the first time you start hopping and jumping and things like that because you've always got that worry in the back of your mind that your knee is going to give way or you're going to re-injure yourself."
4.14	Thoughts about rehab progression and return to preinjury sport/level	Descriptions of participants thoughts about their rehabilitation progression and how this related to returning to their preinjury levels of sport or physical activity	14 references across 6 interviews	"Not yet, I feel like I'm just about ready to although since I've started work I probably have not been doing as much physio as I'd like to be doing normally. So, I think I'm probably at the stage where I could get back into it, it's more just fitting it around my work commitments and everything."
4.15	RTS following previous ACLR (contralateral leg)	Descriptions of returning to sport following previous ACLR on the contralateral side	2 references across 1 interviews	"I was doing it but physically, it wasn't too bad but mentally to get over it, was a lot harder so I didn't push myself too much. I was starting it but I didn't go to that competitiveness zone, I would say, where I was before the surgery. I would say it took me around a year, it did take me a year."

Theme 5: Balancing resources, advice & opinions

5.01	Advice and treatment outside the UK	Descriptions of advice given and treatment received outside of a UK healthcare setting	7 references across 2 interviews	"The first thing, in India, they told me; they said the same advice in terms of it's not a life-saving problem, so, it's not something that needs to be operated on, but however, what they told me was, at your age, we recommend you to get operated, but it is left to your choice."
5.02	Advice given re engaging in physical activity pre surgery	Descriptions of advice given about engaging in physical activity prior to surgery	18 references across 11 interviews	"Doctor Z said I shouldn't run on the road or and just run on the treadmill when I am fit enough"

5.03	Advice given regarding surgery	Descriptions of advice given regarding surgery	47 references across 16 interviews	"There was a lot of mixed messages, and then the knee fellow had said, 'Oh you're not doing a high-level sport, so you probably won't need to.' And then I saw Mr A, and you know when you're going like this to that to this to that in your head as to what's going to happen, I thought I'd just be guided by Mr A and he sort of said, 'If I was you, then I'd be having that repaired'"
5.04	Advice given regarding postoperative rehabilitation	Descriptions of advice given regarding postoperative rehabilitation	8 references across 6 interviews	"They said to me, you have to do all the rehabilitation or the operation won't work, they have said that but they ain't actually said what it is or nothing ... Yes, they said that is the most important bit, the rehabilitation really, that's what I got off him because they can do the surgery and if I do that then it's a waste of time doing the surgery, so, I think that is the more important bit, so, he said
5.05	Advice/information given re RTS	Descriptions of advice given regarding returning to sport	7 references across 6 interviews	"So the aim is to be fighting fit for April next year, so that's about nine months on from the surgery and he [the physio] knows that. And, you know, we have sort of said nine months is a push to get you there for nine months but it has been done"
5.06	Consistency of advice	Descriptions and accounts of the consistency of advice participants received	29 references across 14 interviews	"I think the only thing probably was that I had spoken to Doctor X about obviously netball and said, you know, can I still play netball and he said absolutely not. And then I think when I went to see the physio she said I would still probably be able to play as long as I wasn't twisting and turning as much. And then I kind of said to my mum like what do you think and she was like absolutely not, I don't think that's the best thing to do. So I haven't played at all.
5.07	Resources used (preoperatively)	Descriptions and accounts resources used by participants prior to surgery	27 references across 15 interviews	"I didn't really know what your ACL was to be honest with you, so obviously they showed it to me on the scan. And then I did go home and obviously just had a little look at it and I watched some videos of like people after they had had surgery"
5.08	Thoughts on information/resources available	Participants thoughts about information and resources available	36 references across 11 interviews	"I think online, it does say what ACL means, there is no doubt about it, but what I was expecting, as the indirect resource, the information is everywhere, I don't know which one I should believe and which one I shouldn't believe."

5.09	Consequences of lack of information	Descriptions of the consequences of the lack of information given regarding their condition and treatment	32 references across 9 interviews	"I don't know what I am expecting next because I have kind of been in the dark a bit with it. And I don't know whether I am going to get a call next to just say hey your surgery is on this date or whether I am going to have a consultation with a surgical consultant regarding what's going to happen. Still to this day I don't know whether the plan is kind of keyhole or something more extreme than that. I don't know ... I work as an outdoor pursuits instructor, so the last thing I want if I was to get a call tomorrow saying hey we have got you in for surgery in two weeks' time, it's going to be a six month recovery. I can't do that. After two years of waiting for potentially something to happen with my knee, I would have to turn that down because, you know, I have got a child and I have got a mortgage and I can't just turn down work. I can't just stop working like that"
5.10	Influence and advice of/given by friends and family	Descriptions of the influence of opinions from friends and family in addition to advice given by them	32 references across 14 interviews	"before the surgery my Mum was kind of simply saying don't get it done because it's obviously a surgery it's an invasive kind of procedure. You don't know what the outcome is going to be afterwards. So if it, if I didn't have a physio background, if it was just what other people were telling me and my family I probably would have thought, second thoughts about it."
5.11	Influence of the media	Descriptions of the influence of the media	3 references across 2 interviews	"I am a Liverpool fan. I have seen Van Dyke it took him a year to get back to playing football and he's got the best physio's and best coaches around him in the world, so I am not expecting to be kicking a football any time soon"
5.12	Information/Knowledge = power	Accounts of information and knowledge equating to power	28 references across 11 interviews	"And unfortunately you and I both know, NHS don't give enough information on rehab on the injuries. So yes if I was in a situation where I didn't know what I was doing I would have liked more information on how to do it and with my health as well, access to gyms and stuff like that ... I think knowledge is power"
5.13	Concerns	Descriptions of participants concerns	3 references across 2 interviews	"Yes, you worry, obviously, outcomes was mentioned as well, that I should think about—I was surprised about how many things were mentioned, in the future, what I should expect because of the surgery, because of this injury that I had, that knee replacement itself, I have to expect; I should think of within 30 years or whatever."
