# Supplementary material for: ‘Going through the motions’; a rich account of the complexity of the anterior cruciate ligament reconstruction pathway, a UK qualitative study
Source: BMJ Open. 2024 Sep 17;14(9):e079468. doi: 10.1136/bmjopen-2023-079468 (PMC11409353; doi:10.1136/bmjopen-2023-079468)
Supplement: online supplemental file 4 [file bmjopen-14-9-s004.pdf]

Supplementary File 4 - Concerns and questions raised during the preoperative period

Concerns and questions raised during the preoperative period	Supporting Quote(s)
What surgery am I having?	"I don't know what surgery I am expecting" [P7]<br>"All I knew is that I was going to get two procedures" [P17]
How long until I can drive after surgery?	"Do I know how long I can't drive for? … I know absolutely nothing" [P3]
How long will I need a brace/crutches for?	"Do I know how long I'll be in a leg brace? You know how long on crutches for? I know absolutely nothing" [P3]
How long will the recovery period be?	"Well to explain what the surgery entailed and what your recovery's going to look like" [P4]<br>"it depends what surgery I am expecting depends on what Google is telling me and I don't know what surgery I am expecting. I don't know if it's, as I said Google is telling me how long recovery is for like a small keyhole surgery and what the length of the surgery will be and this and that. But then it's telling me something completely different for a different style of knee surgery to repair it a different way and I don't know which one I am having." [P7]<br>"Because the questions that I was asking the nurse, she could sort of give me an idea but couldn't confirm it, so like things like how long the recovery period would take and things like that." [P8]<br>"I don't even know how long it will take me to heal up, nobody has said ought" [P13]
I shouldn't exercise, it's risky	"I don't like it, well it's more that confidence thing I probably could exercise but it's just the mental blocks like what happens if I fall off, what happens if I do further damage to it and that's a risk I don't want to take" [P5]<br>"I didn't really have the confidence to go and run for example. So I have not really done any running for at least two years. A bit of a confidence thing because my knee has given way pre-surgery a couple of times and it's not a nice feeling, but I also know that it's not stable at all so I haven't even risked it really." [P8]

	"there is not enough information for people and the probability of reoccurrence injury is much higher than I would like it to be because people will rush back to exercise, especially football, rugby or any sport where they have torn their ACL… Avoid things that are going to damage it even more" [P9]
Who can I contact for support?	"Like who do you go to? Do you go the surgeon, do I speak to the receptionist? Do you go to the physio who you have not seen in the last year? Do you go to the GP who you are not going to get seen because GPs are difficult to get hold of at the best of times, never mind if it's not an urgent case where you aren't going to drop dead the next day? So it's like where do you go? You are not being given any information. Do you go back to A&E? A&E is absolutely rammed at the minute with patients left, right and centre" [P8]<br>"knowing that someone could be on the end of a phone would have been great." [P16]
What additional damage could I cause whilst waiting?	"he says, 'Oh, you're at a much higher risk of doing further damage if it's not repaired.' And then they leave you for all that big, long period of time to do that further damage, that then he infers might make repair definitely more difficult, or impossible. Do you know what I mean, so I think that you feel a bit adrift for that." [P4]<br>"I probably could exercise but it's just the mental blocks like what happens if I fall off, what happens if I do further damage to it and that's a risk I don't want to take" [P5]<br>"I have kept myself active. I have kept myself moving, I have kept myself really healthy despite my injury but how much extra damage has that done? Has it done extra damage? I don't know." [P7]
What about re-rupture?	"The only thing I'm worried about is well when I was reading up on the surgery side of things if it snaps again because if you have surgery a second time it's going to give it less chance to heal or

	fail. That is the only thing I am worried about; I've just got to make sure I take good care of it in the recovery process." [P5]
What can/can't I do?	"obviously throughout the year and a half I've been waiting I know what my knee is capable of it just that time period, but I suppose a physio could of helped with that don't do these movements, these movements, these movements, only do these certain movements" [P5]<br>"I had no direction for prehab … can I do this, can I do that, whereas I may have just done it ... I want to push myself as much as possible but you need to tell me where that limit is because otherwise I may get nowhere near it." [P11]<br>"I think it would have been good to have a bit of physiotherapy support, and also, to make sure I was doing the right kind of things to prepare myself for the surgery because I didn't really have any plan before the surgery." [P15]
