# Supplementary material for: ‘Going through the motions’; a rich account of the complexity of the anterior cruciate ligament reconstruction pathway, a UK qualitative study
Source: BMJ Open. 2024 Sep 17;14(9):e079468. doi: 10.1136/bmjopen-2023-079468 (PMC11409353; doi:10.1136/bmjopen-2023-079468)
Supplement: Supplementary file 5 [file bmjopen-2023-079468.reviewer_comments.pdf]

PEER REVIEW HISTORY

BMJ Open publishes all reviews undertaken for accepted manuscripts. Reviewers are asked to complete a checklist review form and are provided with free text boxes to elaborate on their assessment. These free text comments are reproduced below.

ARTICLE DETAILS

**TITLE (PROVISIONAL)**	"Going through the motions"; a rich account of the complexity of the anterior cruciate ligament reconstruction pathway, a UK qualitative study.
**AUTHORS**	Carter, Hayley; Beard, David; Leighton, Paul; Moffatt, Fiona; Smith, Benjamin; Webster, Kate E.; Logan, Phillipa

VERSION 1 - REVIEW

**REVIEWER NAME**	***O'Leary, Shaun***
**REVIEWER AFFILIATION**	University of Queensland
**REVIEWER CONFLICT OF INTEREST**	Na
**DATE REVIEW RETURNED**	26-Jan-2024

**GENERAL COMMENTS**	Generally well put together study and manuscript<br>ABSTRACT<br>Setting – could that be a bit more detailed? Across how many facilities? Specific department?<br>Regarding "Ten participants were awaiting ACLR, six were 3-months post-surgery and two were 1-year post-surgery." What impact does these differences in numbers have on the data richness/consistency over the nominated time periods of the study?<br>INTRODUCTION<br>Regarding "Postoperative perspectives from 6-months to 10-years post-surgery……" Would be good to say what this showed that adds something to why this study was undertaken. Overall the second paragraph lacks a bit of insight to what these previous quantitative and qualitative studies indicated that would further show the need to do this study ie. the absence of knowledge, not just the absence of studies<br>METHODS<br>Regarding recruitment - Could more detail be provided that would describe the sample as to how many NHS facilities they come from etc. Its just that if the results represent experiences from just one facility then that may only be relevant to what happens as one facility and that needs to be transparent.<br><br>RESULTS<br>Page 11 second last paragraph – spell out what HCPs are.<br>Regarding the navigating the treatment pathway. I see there is some discussion around surgical pathway decision making. Was there any details regarding how these individuals were selected for a surgical pathway? Particularly as to how expectations may play a large part in a patients outcomes and cognitions towards management. Was non-surgical care proposed as just as a legitimate option?<br><br>DISCUSSION<br>I generally don't have an issue with how the discussion is set out

	and it provides discussion of the implications of the findings as it should rather than just repeating the results, but I think some of the conclusions need to be softened a bit given that it is one study from one region of the UK in 18 people. Its just that concluding statements like “These issues are more prominent than that previously recognised in the literature.” and inferences that this represents the situation in the NHS, are too matter of fact as this may simply reflect the facilities these 18 patients were from. This is why as stated above how many facilities these participants were recruited from is essential information in the recruitment section as findings may simply reflect poor communication from a surgeon/physio/s at one or two facilities, and poorly represent what experience patinets have at others. This limitation acknowledgement should be upfront in this discussion, not just the last paragraph in the limitations section.

**REVIEWER NAME**	***Røise, Olav***
**REVIEWER AFFILIATION**	University of Oslo, Institute of Clinical Medicine
**REVIEWER CONFLICT OF INTEREST**	Na
**DATE REVIEW RETURNED**	09-Feb-2024

**GENERAL COMMENTS**	Clinical researchers are most concerned with results measured by objective means, functional results and development of radiological osteoarthritis over time. General and specific PROMs have gained increasing interest the last decades, but use of PREMs and qualitative methodology is almost absent.<br><br>It is, therefore, very encouraging to see qualitative work that identifies and documents the challenges associated with logistics and patient pathways, information not disclosed in quantitative studies. On the other hand, it is disheartening to see the patients' experiences of a lack of a systematic approach with an agreed programme for patients with the same type of injury. Based on studies within orthopedic traumatology, we have seen much of the same findings in an ongoing qualitative PhD project from a Scandinavian country(1, 2).<br><br>This is a well written and very interesting paper. In the introduction the authors define the context, the knowledge gap and and objectives of the study.<br><br>Regarding the choice of method, this is a recognised method that is well described and documented. However, I do have some questions when it comes to details that is of importance for replications.<br><br>Results are well presented with representative quotes and illustrations. Discussion is relevant and conclusions are based on results.<br><br>COREQ file has been checked and found adequate filled in. Reference to pages where information can be found in the paper is not correct, but I guess that is due to technical issues.<br><br>Enclosed, find comments, proposals and questions to all parts of the paper that should be commented and responded to. My main concerns are related to methodological details that are lacking for

	replication of the study.<br><br>1. Finstad J, Roise O, Clausen T, Rosseland LA, Havnes IA. A qualitative longitudinal study of traumatic orthopaedic injury survivors' experiences with pain and the long-term recovery trajectory. BMJ Open. 2024;14(1):e079161.<br>2. Finstad J, Roise O, Rosseland LA, Clausen T, Havnes IA. Discharge from the trauma centre: exposure to opioids, unmet information needs and lack of follow up-a qualitative study among physical trauma survivors. Scand J Trauma Resusc Emerg Med. 2021;29(1):121.

VERSION 1 – AUTHOR RESPONSE

Reviewer: 1

ABSTRACT

- Setting – could that be a bit more detailed? Across how many facilities? Specific department?

All participants were recruited from the orthopaedic and physiotherapy waiting lists of one NHS Trust. Participans received rehabilitation across a range of geographical sites in the region, and detailed relating to specific hospital departments were not collected. This detail has been added to the methods and results section (detailed below) and a point has been added to the strengths and limitations sections (page 3) and reads:

"The study population were treated within hospitals in one region of the UK (Midlands). Whilst it is not the aim of qualitative research to be generalisable, these findings may not represent the experiences of those treated in other UK regions and outside of an NHS setting. Further detail regarding specific hospital departments was not collected."

- Regarding "Ten participants were awaiting ACLR, six were 3-months post-surgery and two were 1-year post-surgery." What impact does these differences in numbers have on the data richness/consistency over the nominated time periods of the study?

This has been added to the strengths and limitations section of the abstract which reads (page 3): "There was an inconsistent number of participants at the three identified time-points for the study. There were a greater number of participants at the preoperative time-point which is likely to have resulted in greater richness of data regarding the preoperative pathway than that of the postoperative pathway (particularly at the one-year time point where only two participants were interviewed)."

INTRODUCTION

- Regarding "Postoperative perspectives from 6-months to 10-years post-surgery……." Would be good to say what this showed that adds something to why this study was undertaken. Overall the second paragraph lacks a bit of insight to what these previous

quantitative and qualitative studies indicated that would further show the need to do this study ie. the absence of knowledge, not just the absence of studies

Further detail has been added to elaborate on the need for this study (page 4, paragraph 2): "Collectively, these studies reveal that patients have unrealistic preoperative expectations of returning to physical activity post-surgery and are faced with a post-operative rehabilitation burden that requires an unexpected level of commitment; with participants describing a lack of mental preparation for the rehabilitation process that was longer and more intense than expected.[9,11,12] There is an absence of knowledge to understand participants' lived experience of this phenomena, particularly prior to surgical intervention. Further, there is a paucity of evidence to understand where patients seek healthcare advice following an ACL rupture diagnosis."

METHODS

- **Regarding recruitment - Could more detail be provided that would describe the sample as to how many NHS facilities they come from etc. Its just that if the results represent experiences from just one facility then that may only be relevant to what happens as one facility and that needs to be transparent.**

All participants were recruited from the orthopaedic and physiotherapy waiting list at one hospital trust. Some participants recruited from the orthopaedic waiting list received rehabilitation at another site. The sites at which they received rehabilitation varied across the midlands and detail regarding specific hospital departments was not collected. We have amended this section to clarify this point in the recruitment sections which reads (page 5, paragraph 2 under 'recruitment'): "Participants were identified by the clinician in charge of their care and recruited from physiotherapy and orthopaedic waiting lists at the University Hospitals of Derby and Burton NHS Foundation Trust (UHDB). Orthopaedic waiting lists were screened for two lower limb consultants at UHDB and all outpatient MSK physiotherapists working at the Florence Nightingale Community Hospital. All patients had received an MRI and had a consultation with an orthopaedic clinician to determine the extent of concomitant injuries. Clinicians were aware of the study eligibility criteria and highlighted all those appropriate for inclusion in the study. They were subsequently contacted by mail or telephone or introduced in person to the researcher (HC)." We have also added this point so the discussion section of the manuscript (described below).

RESULTS

- **Page 11 second last paragraph – spell out what HCPs are.**

All HCP abbreviations have been removed and replaced with healthcare professional/s.

- **Regarding the navigating the treatment pathway. I see there is some discussion around surgical pathway decision making. Was there any details regarding how these individuals were selected for a surgical pathway? Particularly as to how expectations may play a large part in a patients outcomes and cognitions towards management. Was non-surgical care proposed as just as a legitimate option?**

Unfortunately, there was no detail as to how participants were selected for the surgical pathway outside that which is described.

DISCUSSION

- I generally don't have an issue with how the discussion is set out and it provides discussion of the implications of the findings as it should rather than just repeating the results, but I think some of the conclusions need to be softened a bit given that it is one study from one region of the UK in 18 people. Its just that concluding statements like "These issues are more prominent than that previously recognised in the literature." and inferences that this represents the situation in the NHS, are too matter of fact as this may simply reflect the facilities these 18 patients were from. This is why as stated above how many facilities these participants were recruited from is essential information in the recruitment section as findings may simply reflect poor communication from a surgeon/physio/s at one or two facilities, and poorly represent what experience patients have at others. This limitation acknowledgement should be upfront in this discussion, not just the last paragraph in the limitations section.

Thank you for highlighting this. The language used has been amended in response to this comment. The highlighted sentence now reads (page 21, 2nd paragraph) "These issues have not previously been recognised in the literature." We have also added a paragraph to the discussion (page 19, 4th paragraph) which reads "It is important to acknowledge that participants in this study were recruited from one hospital Trust but received rehabilitation at a range of different sites within the Midlands. Whilst data on specific sites were not collected, the experiences discussed may not represent that of other areas of the UK."

Reviewer: 2

Abstract

Strengths & Limitations

- Do you think that 3 patients are sufficient for the 1-year postop group.

This was also acknowledged by reviewer 1 and an additional point has been added to the strengths and limitations section of the abstract (page 3) which reads: "There was an inconsistent number of participants at the three identified time-points for the study. There were a greater number of participants at the preoperative time-point which is likely to have resulted in greater richness of data regarding the preoperative pathway than that of the postoperative pathway (particularly at the one-year time point where only two participants were interviewed)." This has also been added to the strengths, limitations and reflexive considerations section of the manuscript (page 20).

Method

Recruitment

- It should be clarified that this is not a longitudinal study by exclusively pinpointing number of patients for the different timepoints as done in abstract. Easy for reader to misunderstand and look at this as en longitudinal study

We have added detail to clarify this and amended the sentence (page 6) to read: "We sought to recruit approximately 12 patients at three separate time-points"

- Refer to table 2 for details!

The section of the manuscript highlighted and referring to this point has been amended to (page 5): "We aimed to include a range of participant characteristics including age, sex, physical activity type and level and prehabilitation engagement (detailed in Table 2 and 3)."

- More information on patient identification by the clinician in charge of care - the procedure is of importance to say anything about skew recruitment of patients. Did the clinician know the patients with detailed information about concurrent joint injuries or was the identification made by random on a patient list without knowledge on injury details?

We have added this information and the paragraph now reads (page 5): "Participants were identified by the clinician in charge of their care and recruited from physiotherapy and orthopaedic waiting lists at the University Hospitals of Derby and Burton NHS Foundation Trust. Clinicians were aware of the study eligibility criteria and highlighted all those appropriate for inclusion in the study."

- Did you include patients from several waiting list or from just one for all the patients? To replicate the study this information is of value. Several hospitals?

- All the included patients were screened with MRI to exclude concomitant injuries requiring surgery? Specify.

Further detail has been added to provide clarity regarding these points. The paragraph (page 5) now reads:

"Participants were identified by the clinician in charge of their care and recruited from physiotherapy and orthopaedic waiting lists at the University Hospitals of Derby and Burton NHS Foundation Trust (UHDB). Orthopaedic waiting lists were screened for two lower limb consultants at UHDB and all outpatient MSK physiotherapists working at the Florence Nightingale Community Hospital. All patients had received an MRI and had a consultation with an orthopaedic clinician to determine the extent of concomitant injuries. Clinicians were aware of the study eligibility criteria and highlighted all those appropriate for inclusion in the study. They were subsequently contacted by mail or telephone or introduced in person to the researcher (HC)."

Data Collection

- Number of participant are described in the abstract - this information should also be given in the method section - even with more details on age (mean values and range), female/male ratio, activity level and type before inclusion and other demographics.

This information is provided in the findings section (page 8) which reads: "Participant characteristics are shown in Table 2. Participants ranged from 18 to 45 years of age (median: 29 years), three identified as female (16.7%) with the remainder identifying as male (83.3%). Ethnic origin of participants was predominantly white (n=14, of which one participant also described themselves as Lithuanian), followed by Indian (n=2), British Asian (n=1) and Pakistani (n=1). Ten participants were awaiting ACLR, six were 3-month post-surgery and two

were 1-year post-surgery. Injuries were sustained predominantly during a sporting activity (n=12) with the remainder occurrig from a slip (n=2), landing from a jump (n=2), a motorcycle incident (n=1) and road traffic collision (n=1). All participants engaged in more than one activity type prior to injury, with 22 different types reported. Football was the most common activity (n=8), followed by attending the gym for resistance training alone (n=7) or cardiovascular and resistance training (n=5), swimming (n=4), rugby (n=3), running (n=3) and cricket (n=3). Other activity types are shown in Table 2. The physical activity types participants were engaged in at the time of interview spanned a smaller variety with resistance training the most commonly reported activity (n=7) followed by walking (n=5) and cardiovascular and resistance training at a gym (n=5). Activity types are shown in Table 3. Twelve participants engaged in prehabilitation (varied treatment length, type, frequency) at different sites across the midlands. The average number of days participants where physically active prior to injury was 4.5 days compared with 3.4 days at the time of interview."

- Was part of the study undertaken during the pandemic? Anyway- offer a sentence telling when the interviews were done (date to date).

Thank you for highlighting this consideration, we have added the interview date range to the data collection paragraph (page 6) which now reads "Participants were offered choice of interview location and medium (face-to-face/telephone/video). Eight participants opted for a face-to-face interview conducted in a hospital setting and the remainder opted for a telephone interview. Interviews were carried out between August and November 2022." and a section under Theme 2 (page 12) acknowledging the impact of COVID19 which reads: "An important factor to consider, impacting patient experience on the surgical pathway, is the timing of this research study. Interviews were carried out two and a half years after the COVID-19 pandemic reached the UK. COVID-19 had a profound impact on NHS services with many face-to-face procedures delayed and the rapid implementation of virtual consultations.[20] Although the NHS recovery plan for tackling the backlog of elective procedures was rolled out in February 2022,[21] service demand and capacity remained unevenly balanced. Unsurprisingly, many participants referenced COVID-19 to justify short falls in their care, it was the predominant reason referenced for the delay to surgery with participants offering some leniency because of this. Participants also reflected on the priority of their injury in comparison to others requiring medical attention during the pandemic. Understanding capacity within the hospital was typically measured against reports in the media, with participants starting to avoid attending the hospital when, perhaps, prior to COVID-19 they may have presented sooner."

- "consent" I guess written - should be specified.

The sentence now reads: "Written consent was taken prior to the interview and recording."

Data Analysis

- Who did this review - control of transcription

This detail has been added to clarify that transcriptions were reviewed by HC (page 6).

Findings

- This information is not in line with the information given in the Recruitment section - see comments on that as well and give more detailed information in the Recruitment section.

The methods and recruitment section has been amended to add further detail and now reads (page 8, paragraph 1):

"A purposive sample of 18 participants were recruited from physiotherapy and orthopaedic waiting and clinic lists at the University Hospitals of Derby and Burton NHS Foundation Trust. Some participants recruited from the orthopaedic waiting list received rehabilitation (pre and/or post) at another site. The sites at which they received rehabilitation varied across the Midlands and detail regarding specific hospital departments was not collected."

- Physically active is a pretty wide concept - can this be elaborated?

Further detail regarding physical activity has been added and addressed in detail in the following points.

- Table 2:
Informative table - information on type of preinjury dominant activity is missing. The information was not collected?

The information in the table should be elaborated in the table text or better in a paragraph of the results (How many had rehab, regained activity level in the different patients group . A short summary).

Information regarding the types of physical activity the participant was engaged in prior to injury and that which they were engaged in at the time of the interview has been added in Table 3 (page 10). The dominant activity type was not collected. Further information has been added to the main body of text (page 8, 2nd paragraph) which now reads:

"Interview length was 25-51 minutes (mean: 38 mins). Participants ranged from 18 to 45 years of age (median: 29 years), three identified as female (16.7%) with the remainder identifying as male (83.3%). Ethnic origin of participants was predominantly white (n=14, of which one participant also described themselves as Lithuanian), followed by Indian (n=2), British Asian (n=1) and Pakistani (n=1). Ten participants were awaiting ACLR, six were 3-month post-surgery and two were 1-year post-surgery. Injuries were sustained predominantly during a sporting activity (n=12) with the remainder occurring from a slip (n=2), landing from a jump (n=2), a motorcycle incident (n=1) and road traffic collision (n=1). Participants engaged in a range of different activity types prior to injury. Football was the most common activity (n=9), followed by attending the gym for resistance training alone (n=7) or cardiovascular and resistance training (n=5), swimming (n=4), rugby (n=3) and running (n=3). Other activity types are shown in Table 2. The physical activity types participants were engaged in at the time of interview spanned a smaller variety with resistance training the most commonly reported activity (n=8) followed by walking (n=5) and cardiovascular and resistance training at a gym (n=4). Twelve participants engaged in prehabilitation (varied treatment length, type, frequency). The average number of days participants where physically active prior to injury was 4.5 days compared with 3.4 days at the time of interview."

Theme 1

- Can you say anything about differences among the groups following different pathways?

- "HCP": Annoying as reader not being able to find out what the abbreviation means without going back trying to find it - why not write it out completely? I guess it is health care personnel.

All HCP abbreviations have been removed and replaced with healthcare professional/s.

- "It was felt that mental wellbeing was not addressed by clinicians; despite musculoskeletal and orthopaedic injuries being commonly linked to poorer mental health outcomes.[19]" This is more of a discussion point - use of references should be avoided in result section.

This discussion point has been moved to the clinical implications section of the discussion (page 19, 2nd paragraph) which reads: "Further, it was felt that mental wellbeing was not addressed by clinicians; despite musculoskeletal and orthopaedic injuries being commonly linked to poorer mental health outcomes.19 This could also be considered by clinicians to ensure appropriate support and sign-posting is provided to patients to manage their condition and make decisions about their care."

Discussion

- "exploration of patient experiences of the NHS ACLR pathway." but not the first qualitative study on postoperative experiences as referred in your introduction - therefore, nuance this sentence.

This sentence has been amended and now reads (page 18, 1st sentence): "This study is the first qualitative exploration of patient experiences specific to the NHS ACLR pathway."

- "NHS" I know what NHS abbreviation is - but write it out in full the first time it is used - also for HCP

The abbreviations for NHS and HCP have been expanded.

VERSION 2 – REVIEW

**REVIEWER NAME**	***O'Leary, Shaun***
**REVIEWER AFFILIATION**	University of Queensland
**REVIEWER CONFLICT OF INTEREST**	Na
**DATE REVIEW RETURNED**	20-Mar-2024

**GENERAL COMMENTS**	First of all, I would say that the authors have made important changes to the article and thereby raised the quality of the paper. New Table 3 clarifies what the patient could do before and after the injury/treatment.<br><br>With regard to my comments and objections, the authors have complied with all input from the undersigned. Upon re-examination of the manuscript with amendments, I have only a few comments; The main one is that they have included a section on page 12 where they have important comments on purely contextual aspects of the study (Covid and effects on waiting lists in the NHS). I think the message they are making is important and raises the quality of the pzper, but I think this fits better in the discussion section than in the results.<br><br>Otherwise, I have noted that they use the term "resistant training" where I myself would have used "muscle strict training", but here I may well have been wrong as not native English speaking, so this is therefore just a question.<br><br>Finally, I have a linguistic suggestion for correction in the last sentence on page 4 marked in yellow; Further detail regarding specific hospital departments was not collected. Change to; Further details.....<br><br>See also the draft that I have uploaded with my comments.

VERSION 2 – AUTHOR RESPONSE

Reviewer: 2

First of all, I would say that the authors have made important changes to the article and thereby raised the quality of the paper. New Table 3 clarifies what the patient could do before and after the injury/treatment. With regard to my comments and objections, the authors have complied with all input from the undersigned. Upon re-examination of the manuscript with amendments, I have only a few comments; The main one is that they have included a section on page 12 where they have important comments on purely contextual aspects of the study (Covid and effects on waiting lists in the NHS). I think the message they are making is important and raises the quality of the paper, but I think this fits better in the discussion section than in the results.

Thank you for the suggestion to move the paragraph relating to COVID-19 on page 12. We have left the section of the paragraph detailing participants descriptions of COVID-19 in Theme 2, as we believe this reflects 'findings of the interviews as participants accounted their experiences of care during COVID-19. On reflection, we agree that part of the paragraph fits better in the discussion. We have therefore deleted the section discussing the contextual factors of COVID-19 and moved this to the strengths, limitations and reflexive considerations section.

Page 12 (findings), paragraph 3 now reads:

“Unsurprisingly, many participants referenced COVID-19 to justify shortfalls in their care. It was the predominant reason referred to for the delay to surgery with participants offering some leniency because of this. Participants also reflected on the priority of their injury in comparison to others requiring medical attention during the pandemic. Understanding capacity within the hospital was typically measured against reports in the media, with participants starting to avoid attending the hospital when, perhaps, prior to COVID-19 they may have presented sooner.”

Page 19 (discussion), paragraph 3 now reads:

“Another important factor to consider, impacting patient experience on the surgical pathway, is the timing of this research study. Interviews were carried out two and a half years after the COVID-19 pandemic reached the UK. COVID-19 had a profound impact on NHS services with many face-to-face procedures delayed and the rapid implementation of virtual consultations [19]. Although the NHS recovery plan for managing the backlog of elective procedures was rolled out in February 2022 [20] service demand and capacity remained unevenly balanced. Unsurprisingly, several participants attributed short falls in their care to COVID-19.”

Otherwise, I have noted that they use the term "resistant training" where I myself would have used "muscle strict training", but here I may well have been wrong as not native English speaking, so this is therefore just a question.

Thank you for raising this question about the terminology used. We have amended resistance training to muscle strength training as suggested in this comment and annotated on the manuscript. The text now reads (page 8, paragraph 2):

“Football was the most common activity (n=8), followed by attending the gym for muscle strength training alone (n=7) or cardiovascular and muscle strength training (n=5), swimming (n=4), rugby (n=3), running (n=3) and cricket (n=3). Other activity types are shown in Table 3. The type of physical activity participants were engaged in at the time of interview spanned a smaller variety with muscle strength training the most commonly reported activity (n=7) followed by walking (n=5) and cardiovascular and muscle strength training at a gym (n=5).”

This has also been amended in Table 3.

Finally, I have a linguistic suggestion for correction in the last sentence on page 4 marked in yellow; Further detail regarding specific hospital departments was not collected. Change to; Further details.....

Thank you for the suggestion, however, in response to the editor’s first comment, we have deleted this sentence.

See also the draft that I have uploaded with my comments.

Thank you for uploading an annotated version of the manuscript. We have reviewed this and believe we have responded to both comments in the above points about the terminology used to describe muscle strength training and moving the COVID-19 paragraph.
